# Acute Liver Failure Requiring Liver Transplantation due to Acute Hepatitis A Virus Infection

**DOI:** 10.1155/2021/5159934

**Published:** 2021-12-27

**Authors:** Chencheng Xie, Jonathan M. Fenkel, Dina L. Halegoua-DeMarzio, Jesse M. Civan, Danielle M. Tholey, Steven K. Herrine, Manish Thapar, Manju Ambelil, Sanaa Arastu, Adam M. Frank, Ashesh P. Shah, Jamie M. Glorioso, Carlo G. Ramirez, Adam S. Bodzin, Warren R. Maley, David A. Sass

**Affiliations:** ^1^Division of Gastroenterology & Hepatology, Thomas Jefferson University, USA; ^2^Department of Pathology, Thomas Jefferson University, USA; ^3^Division of Transplant Surgery, Thomas Jefferson University, USA

## Abstract

**Introduction:**

Hepatitis A infection (HAV) is generally characterized by an acute icteric illness or may have a subclinical self-limited course, although rarely, can result in fulminant hepatitis and death. In 2019, the City of Philadelphia declared a public health emergency due to an HAV outbreak. We are reporting a series of four cases of acute liver failure (ALF) requiring liver transplantation (LT) due to acute HAV.

**Methods:**

Chart review and case descriptions of four patients with acute HAV-related ALF who were expeditiously evaluated, listed as Status 1A, and who underwent LT between August 2019 and October 2019 at Thomas Jefferson University Hospital.

**Results:**

All four patients presented with acute hepatocellular jaundice and had a positive HAV IgM, and all other causes of ALF were excluded. All four cases met the American Association for the Study of Liver Diseases (AASLD) criteria for ALF. Three of the four cases met King's College Criteria of poor prognosis for nonacetaminophen-induced ALF. All four patients underwent successful LT and were discharged six to twelve days postoperatively. One patient died of disseminated Aspergillus infection five months after LT, while the others have had excellent clinical outcomes shown by one-year follow-ups. All four explants had remarkably similar histological changes, revealing acute hepatitis with massive necrosis accompanied by a prominent lymphoplasmacytic inflammatory infiltrate and bile ductular proliferation.

**Conclusion:**

Although rare, patients presenting with acute HAV need close monitoring as they may rapidly progress to ALF. Early referral to a transplant center afforded timely access to LT and yielded overall good one-year survival. Widespread HAV vaccination for high-risk individuals is an essential strategy for preventing disease and curbing such future outbreaks.

## 1. Introduction

The primary transmission route of HAV is the fecal-oral route via either person-to-person contact or via contaminated food or water. With programs distributing vaccinations to high-risk individuals, HAV incidence has declined by approximately 95% from 1996 to 2011. However, the Centers for Disease Control and Prevention (CDC) reported that HAV reached about 15,000 cases during the period from 2016 to 2018. The cases increased by 294% compared with the period from 2013 to 2015, indicating outbreaks in the United States of America [1]. Because of its mode of transmission, acute HAV can present as an epidemic in resource poor settings. In 2019, the Philadelphia Department of Public Health (PDPH) declared a public health emergency response to rapid increases in new HAV cases. From January through December 2019, there were four hundred and twenty-six outbreak-related cases, including four deaths, in Philadelphia. In comparison, there were approximately twenty HAV cases in the city in 2018 [2]. HAV is characterized by an acute and often self-limited (subclinical) disease course, which rarely causes fatal clinical consequences. It is estimated that less than 1% of patients with acute HAV can develop ALF, and about one-third of these require LT [3, 4]. Due to the rarity of such cases, we summarized the case series of ALF requiring LT at our quarternary care, inner city, academic medical center.

## 2. Methods

Our team retrospectively reviewed charts at Thomas Jefferson University Hospital, a high-volume academic transplant center. Four cases of acute HAV-ALF, who were carefully evaluated and listed as Status 1A, were identified, and all four cases underwent LT between August 2019 and October 2019. Charts were further reviewed for details, and data on the demographics, laboratory results, imaging, preoperative conditions, time from admission to transplantation, and explant pathology were collected.

## 3. Results

There were one male and three female patients between the ages of 44 and 69 years of age. Patients reported feeling unwell with an abrupt symptom onset of nausea, vomiting, diarrhea, chills, fever, body aches, poor appetite, and fatigue from one week to one month before admission. All four cases had marked liver transaminase elevation with positive HAV IgM, while all other causes of acute liver injury were excluded.

### 3.1. Patient #1

Patient #1 was a 64-year-old female with a history of remote alcohol dependence but no advanced liver disease. Her initial presentation was abdominal pain and mild jaundice, and she was noted to have marked hepatocellular liver injury. Her model for end-stage liver disease (MELD) score reached 40. She met the AASLD criteria for ALF [5] and King's College Criteria for poor prognosis for nonacetaminophen-induced ALF [6]. She was urgently listed as Status 1A and received a LT one day after admission. She was noted to have focal ischemic colitis with mucosal necrosis in the cecum and proximal ascending colon during the transplant operation. Hence, a right hemicolectomy with primary ileocolostomy was performed during the surgery. Her explant showed acute hepatitis with massive hepatic necrosis. Her post-LT course was further complicated by failure to thrive, a de novo cytomegalovirus (CMV) viremia at two months following LT, as well as a late-onset (>90 days post LT) systemic invasive *Aspergillus fumigatus* infection with spinal, renal, and cardiac involvement. Despite immunosuppression withdrawal and aggressive medical and antifungal therapy, she died five months after liver transplantation of the invasive Aspergillus infection.

### 3.2. Patient #2

Patient #2 was a 69-year-old female with a history of daily alcohol use of three glasses of spirits. Previous ultrasonography had indicated hepatic steatosis. She presented with abdominal pain, nausea, vomiting, and jaundice as well as marked transaminitis and acute kidney injury. Her MELD score was 40 at presentation, she was encephalopathic, and she met the AASLD criteria for ALF and King's College Criteria for poor prognosis. She was listed as a Status 1A and received a LT within one day of listing. She had an uneventful postoperative course and was discharged six days after LT. Her explant also demonstrated acute hepatitis with massive hepatic necrosis. She continues to do well twenty-two months post-LT with normal allograft function on tacrolimus monotherapy.

### 3.3. Patient #3

Patient #3 was a 44-year-old female who only drank alcohol socially, about one to two drinks each week, and did not have any preexisting chronic liver disease. Her presentation was that of jaundice, generally feeling unwell with laboratory studies demonstrating hepatocellular liver injury and severe coagulopathy. Her MELD score was 39 on admission, and she met the AASLD criteria for ALF but not King's College Criteria for poor prognosis. She was listed as a Status 1A and received LT within one day of listing. Her explant again demonstrated an acute hepatitis picture with massive hepatic necrosis. She was discharged seven days postoperatively after an uneventful course. At eight months post-LT, she developed a low-level CMV viremia, and an allograft biopsy showed hepatic granulomas. She is now twenty-three months post-LT and doing very well on tacrolimus monotherapy and is no longer requiring suppressive antiviral agents for her CMV.

### 3.4. Patient #4

Patient #4 was a 64-year-old male with a history of HIV (stable on highly active antiretroviral therapy (HAART) for three years) but no preexisting chronic liver disease. He drank alcohol socially, about one to two cans of beer each week. His presenting symptoms were several days of malaise, chills, fevers, body aches, and jaundice, and his admission laboratory tests showed marked hepatocellular liver injury and coagulopathy. He had a MELD score of 40 and met both the AASLD criteria for ALF and King's College Criteria for poor prognosis. He was listed as a Status 1A and received LT within one day of listing. His explant showed acute hepatitis with massive hepatic necrosis. He was discharged twelve days after LT without complications. He is doing very well twenty-two months post-LT on stable-dose tacrolimus monotherapy.

**Figure 1:**
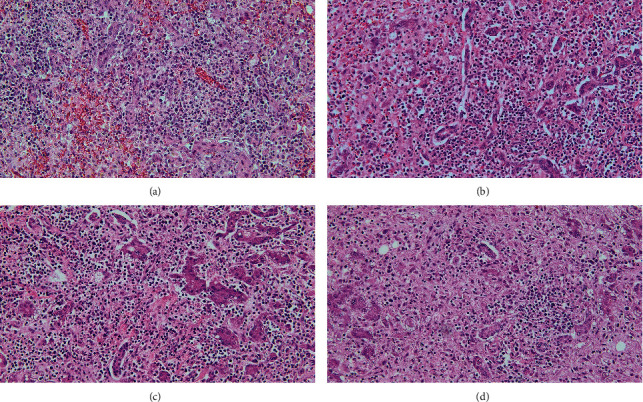
Liver explant pathology of HAV-ALF: all four patients presented similar histological changes. H&E stain shows massive hepatocellular necrosis with prominent lymphoplasmacytic inflammatory infiltration in (a–d) (magnification ×20).

Table 1 summarizes the key demographic, clinical, laboratory, and explant pathology data of the four cases. All of them had peak aspartate aminotransferase (AST) and alanine aminotransferase (ALT) above 5000 IU/L, total bilirubin between 6.7 and 13 mg/dL, international normalized ratio (INR) above 6, and their conditions were complicated by acute kidney injury during the hospitalization. All explants were remarkably similar histologically, showing acute hepatitis with massive hepatic necrosis accompanied by a prominent lymphoplasmacytic inflammatory infiltrate and bile ductular proliferation (see Figures 1(a)–1(d)). While one patient died of a disseminated *Aspergillus* infection five months after LT, the others have had excellent one-year clinical outcomes. All patients were transferred to Thomas Jefferson University Hospital from other hospitals, and three of the four originated outside the Philadelphia City limits.

## 4. Discussion

The CDC's annual surveillance data indicated a relatively stable HAV infection rate between 2012 and 2015, which was only 0.4 per 100,000 of the population. However, a surge of 44.4% in HAV infections was identified in 2016. The CDC issued a nationwide HAV outbreak due to the increasing number of new cases and rapidly soaring number of HAV hospitalizations [1, 7]. The PDPH also declared a public health emergency in response to the alarming number of new HAV cases in Philadelphia in August 2019. Among the four hundred and twenty-six HAV outbreak-related cases in Philadelphia, 60% were linked to illicit drug use and homelessness; other high-risk groups including men who have sex with men and people who are currently, or were recently, incarcerated. However, 35% of cases had no known risk factors for the acquisition of HAV [2]. In our case series, only one patient, who had chronic HIV, had identifiable risk factors for HAV acquisition. Only one of four patients used recreational marijuana, but no others were active drug users, and none were experiencing homelessness.

Vaccination has dramatically reduced acute HAV infections by 67% in the United States. This reflects the impact that public health initiatives can have on curbing HAV outbreaks [8, 9]. The PDPH's efforts in delivering vaccines to high-risk populations have proven to be an effective strategy for disease control. As HAV vaccinations increased among the target population, HAV infections decreased by more than 90% in four months, compared with the peak in Philadelphia [2].

In general, less than 1% of HAV infections result in ALF. Meanwhile, HAV only accounts for 3% of ALF in the United States [3, 10]. HAV-related ALF usually has an overall good prognosis. 70% of HAV-related ALF can spontaneously recover with supportive care but about 30% need LT to survive [4]. Fujiwara et al. conducted a polygenetic analysis on twenty-five HAV-infected patients, including seven cases of fulminant hepatitis, five cases of severe acute hepatitis, and thirteen cases of self-limited acute hepatitis, and identified that genetic variations in the HAV 5′ nontranslated region (5′NTR) and nonstructural proteins 2B and 2C might cooperatively influence HAV replication and further affect virulence [11]. Also, it has been noticed that cytolytic T cells may play a critical role in the pathogenesis among severe HAV cases [12]. There might have been multiple aggravating factors contributing to the critically ill conditions in our HAV-ALF case series including this being a more virulent strain of the HAV virus. A sequencing and genetic variations analysis might help decode these factors for future studies.

It was estimated that acute kidney injury (AKI) occurred in about 3-5% of HAV infection cases in two large retrospective cohorts and was associated with risk factors of alcohol, diabetes, high levels of transaminase and bilirubin, and prolonged hospital stay [1, 13]. Among HAV-related death cases, 12.7% were attributed to AKI according to the United States National Vital Statistics System Multiple Cause of Death data in 2011 [14]. In our series, all four patients' cases were complicated by AKI. Renal insufficiency in ALF requiring LT might be more frequent than previously thought.

One patient died due to invasive aspergillosis (IA) five months after LT. She had multiple risk factors for IA include immunosuppression, renal insufficiency, and CMV infection. As previously outlined, the other three cases have had excellent one-year clinical outcomes.

## 5. Conclusion

Although rare, acute HAV may result in ALF and patients need to be closely monitored. Early recognition of ALF and urgent referral to a transplant center affords timely access to LT, and overall, the posttransplantation one-year survival rate is very good. Vaccination is recommended for adults who are at high risk including illicit drug users, those suffering from homelessness, men who have sex with men, those who are dealing with incarceration, those with chronic liver disease, and persons who plan to travel to HAV-endemic countries.

## Figures and Tables

**Table 1:** Summary of four cases of Acute HAV-related ALF requiring LT.

Patient	Age/gender	Lab tests					MELD	HE	Status 1A (Y/N)	Time from admission to LT (days)	Pathology
		pAST (IU/L)	pALT (IU/L)	pT-bili (mg/dL)	pINR	pCr (mg/dL)					
1	64 F	7471	7270	6.7	>8.18	2.0	40	Y	Y	1	Acute hepatitis with massive hepatic necrosis
2	69 F	5442	5123	10.6	8.31	1.96	40	Y	Y	2	Acute hepatitis with massive hepatic necrosis
3	44 F	7500	6690	9.2	6.33	1.5	39	Y	Y	2	Acute hepatitis with massive hepatic necrosis
4	64 M	9137	9021	13.0	8.04	2.14	40	Y	Y	3	Acute hepatitis with massive hepatic necrosis

F: female; M: male; pAST: peak aspartate aminotransferase; pALT: peak alanine aminotransferase; pT-bili: peak total bilirubin; pINR: peak international normalized ratio; pCr: peak creatinine; MELD: model for end-stage liver disease score; HE: hepatic encephalopathy; Y: yes; N: no; LT: liver transplantation.

## Data Availability

The data used to support the findings of this study are included within the article.
